# Placental Vesicles Carry Active Endothelial Nitric Oxide Synthase and Their Activity is Reduced in Preeclampsia

**DOI:** 10.1161/HYPERTENSIONAHA.117.09321

**Published:** 2017-07-12

**Authors:** Carolina Motta-Mejia, Neva Kandzija, Wei Zhang, Vuyane Mhlomi, Ana Sofia Cerdeira, Alexandra Burdujan, Dionne Tannetta, Rebecca Dragovic, Ian L. Sargent, Christopher W. Redman, Uday Kishore, Manu Vatish

**Affiliations:** From the Nuffield Department of Obstetrics & Gynaecology, University of Oxford, Women’s Centre, John Radcliffe Hospital, United Kingdom (C.M.-M., N.K., W.Z., V.M., A.S.C., A.B., R.D., I.L.S., C.W.R., M.V.); Biosciences, College of Health and Life Sciences, Brunel University London, Uxbridge, United Kingdom (C.M.-M., U.K.); and Department of Food and Nutritional Sciences, University of Reading, United Kingdom (D.T.).

**Keywords:** endothelial nitric oxide synthase, hypertension, nitric oxide, preeclampsia, syncytiotrophoblast extracellular vesicles

## Abstract

Preeclampsia, a multisystem hypertensive disorder of pregnancy, is associated with increased systemic vascular resistance. Placentae from patients with preeclampsia have reduced levels of endothelial nitric oxide synthase (eNOS) and, thus, less nitric oxide (NO). Syncytiotrophoblast extracellular vesicles (STBEV), comprising microvesicles (STBMV) and exosomes, carry signals from the syncytiotrophoblast to the mother. We hypothesized that STBEV-bound eNOS (STBEV-eNOS), capable of producing NO, are released into the maternal circulation. Dual-lobe ex vivo placental perfusion and differential centrifugation was used to isolate STBEV from preeclampsia (n=8) and normal pregnancies (NP; n=11). Plasma samples of gestational age–matched preeclampsia and NP (n=6) were used to isolate circulating STBMV. STBEV expressed placental alkaline phosphatase, confirming placental origin. STBEV coexpressed eNOS, but not inducible nitric oxide synthase, confirmed using Western blot, flow cytometry, and immunodepletion. STBEV-eNOS produced NO, which was significantly inhibited by *N* ^G^-nitro-l-arginine methyl ester (eNOS inhibitor; *P*<0.05) but not by *N*-(3-(aminomethyl) bezyl) acetamidine) (inducible nitric oxide synthase inhibitor). STBEV-eNOS catalytic activity was confirmed by visualizing eNOS dimerization. STBEV-eNOS was more abundant in uterine vein compared with peripheral blood, indicating placental origin. STBEV isolated from preeclampsia-perfused placentae had lower levels of STBEV-eNOS (STBMV; *P*<0.05) and overall lower NO activity (STBMV, not significant; syncytiotrophoblast extracellular exosomes, *P*<0.05) compared with those from NP. Circulating plasma STBMV from preeclampsia women had lower STBEV-eNOS expression compared with that from NP women (*P*<0.01). This is the first observation of functional eNOS expressed on STBEV from NP and preeclampsia placentae, as well as in plasma. The lower STBEV-eNOS NO production seen in preeclampsia may contribute to the decreased NO bioavailability in this disease.

## Abstract 2

Supplemental Digital Content is available in the text.

Early in normal pregnancy (NP), maternal blood volume expands while systemic vascular resistance and systemic blood pressure both decline.^1^ These changes alter significantly in preeclampsia, a pregnancy-specific syndrome (affecting 5% to 10% of all pregnancies worldwide),^2^ which is defined by maternal new-onset hypertension and proteinuria or organ dysfunction, developing after 20 weeks of gestation.^3^ Preeclampsia is thought to originate as a result of poor placentation, causing endothelial dysfunction, disordered angiogenic balance and resultant hypertension, glomerular lesions, and hepatic failure.^4,5^

Nitric oxide (NO) is a potent vasodilator, considered to have major effects on gestational endothelial function.^6^ NO is synthesized by nitric oxide synthases (NOS), namely endothelial NOS (eNOS), inducible NOS (iNOS), and neuronal NOS.^7^ Studies investigating circulating levels of NO in preeclampsia have reported conflicting results.^8^ This is in contrast to studies that have shown that plasma from women with preeclampsia elicits reduced endothelium-dependent vasodilatation in isolated vessels. NO availability may be decreased^9–11^ because of oxidative stress, vascular endothelial growth factor deficiency, or endogenous inhibitors, such as asymmetric dimethylarginine.^12^ It is assumed that most of the circulating NO derives from maternal endothelium. But the placenta may also contribute.

In the placenta, NOS is predominantly expressed in the syncytiotrophoblast, villous endothelium, and macrophages; the predominant isoform being eNOS.^13–15^ The multinucleated syncytiotrophoblast layer lining the chorionic villi is the interface between the maternal and fetal vascular systems^16,17^ and could contribute to circulating NO. Here we investigate another possibility, namely, that syncytiotrophoblast-derived NOS could be exported to the mother in syncytiotrophoblast extracellular vesicles (STBEV) with the potential of systemically modulating maternal vascular response. STBEV are shed into the maternal circulation, detectable from week 10 of gestation and in increasing amounts through pregnancy.^18,19^ STBEV comprise 2 subgroups: microvesicles (STBMV, 100–1000 nm), which are shed directly from the plasma membrane in response to cell activation or death, and exosomes (STBEX, 20–200 nm), which are released by exocytosis from multivesicular bodies of the endosome.^20^ There is evidence that STBEV could allow communication between the mother and the fetus, in a manner dependent on the content of their cargo.^21^

Our hypothesis was whether STBEV might carry eNOS as part of their cargo. To investigate this, we examined whether eNOS was present on STBEV derived from NP and preeclampsia-perfused placental lobules, as well as circulating STBMV from NP and preeclampsia peripheral vein blood (PB) and uterine vein blood (UV). We wanted to assess whether STBEV-bound eNOS (STBEV-eNOS) was functional and capable of producing NO, and whether its expression and activity was altered in preeclampsia.

## Methods

### Human Subjects

This project was approved by the Central Oxfordshire Research Ethics Committee C (REFS 07/H0607/74 & 07/H0606/148). All mothers undergoing elective caesarean section gave written informed consent for the use of their blood and placentas. Blood samples were collected in four 5-mL EDTA/citrate tubes using a 21-gauge needle. Samples were centrifuged at 1500*g* for 15 minutes at room temperature, and the supernatant was centrifuged again at 13 000*g* for 2 minutes to produce platelet-free plasma and stored in aliquots at −80°C. PB samples were from the left antecubital fossa, while UV samples were taken at caesarean section (after bladder reflection before the uterine incision was made). Placentae were collected within 10 minutes of delivery. Placentae from NP were from singleton pregnancies with no current or previous history of preeclampsia or hypertensive disorders. Preeclampsia patients were identified according to the criteria of the International Society for the Study of Hypertension in Pregnancy; hypertension was defined as new hypertension in the second half of pregnancy >140/90 mm Hg accompanied with new proteinuria >300 mg per 24 hours. Clinical characteristics of preeclampsia patients and controls (NP) are described in Table. STBMV from plasma samples were matched for gestational age.

**Table.**
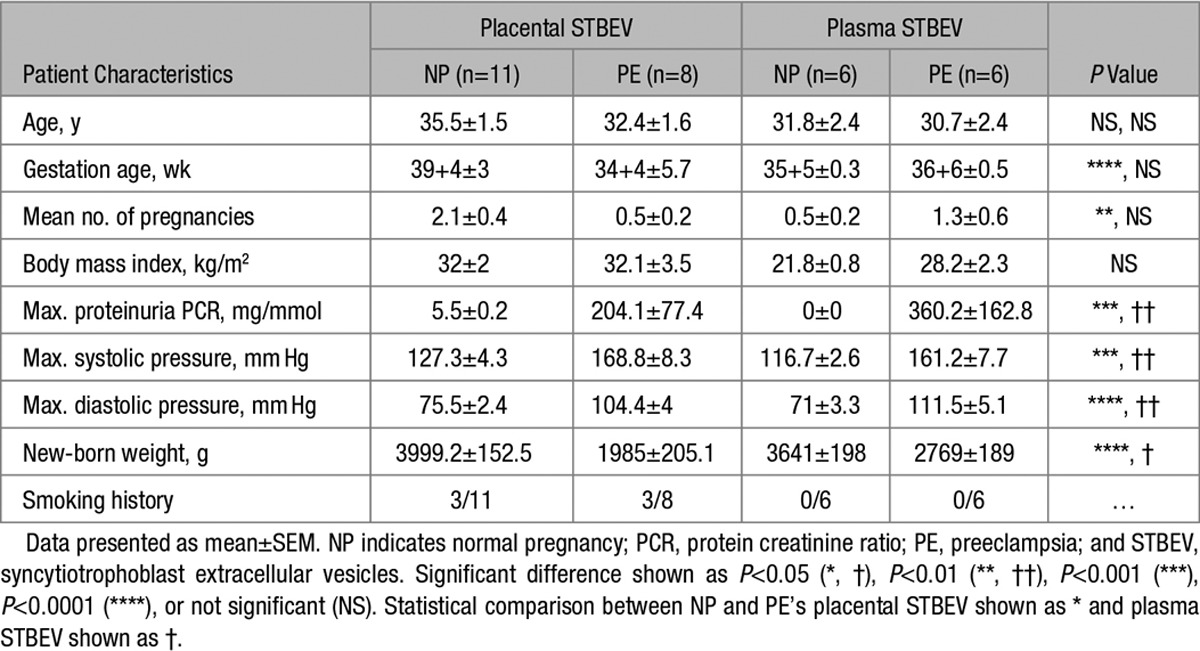
Clinical Data of Human Subjects

### Cell Culture

Human umbilical vein endothelial cells were used as eNOS-positive control. Detailed Methods section is available in the online-only Data Supplement.

### Immunohistochemistry

Immunohistochemical staining was performed using standard protocols with NP placental tissue slide preparations. Detailed Methods section is available in the online-only Data Supplement.

### Isolation and Characterization of STBEV

STBEV were prepared using a modified dual-lobe placental perfusion system and differential centrifugation as previously described by us.^22^ Fresh STBMV were phenotyped by flow cytometry, using a BD LSRII flow cytometer (BD Biosciences), and STBEX were phenotyped by nanoparticle tracking analysis, using a NanoSight NS500 (Malvern, UK). The phenotype of STBEV was additionally assessed by SDS-PAGE and Western blotting analysis for placental alkaline phosphatase (PlAP) positivity (PlAP is syncytiotrophoblast specific) combined with the exosome markers ALIX, Syntenin, and CD9 to confirm exosomal phenotype because STBEX cannot be resolved by flow cytometry because of their size. STBMV and STBEX were assayed for protein using bicinchoninic acid assay before storage at −80°C.

### Flow Cytometry Analysis

Perfusion-isolated STBMV from NP and preeclampsia placentae were interrogated by multicolor flow cytometry using the protocol and settings previously described^22,23^ and specifically interrogated for eNOS. Detailed Methods section is available in the online-only Data Supplement.

Circulating plasma STBMV from PB and UV were analyzed by flow cytometry using a previously described protocol,^24^ with additional modifications to exclude potential nonplacental extracellular vesicle (EV) contaminants. Detailed Methods section is available in the online-only Data Supplement.

Flow cytometry data were analyzed using FACSDiva software 8.0 (BD Biosciences), and figures were generated using FlowJO version 10 (Tree Star Inc, Ashland, OR). Given the issues described above about STBEX and flow cytometry, we investigated STBEV coexpression of eNOS and PlAP using paramagnetic immunobead depletion and Western blotting, described below.

### Western Blotting

Western blots were performed using standard protocols with placental lysate, along with isolated STBMV and STBEX from NP and preeclampsia placentae. Detailed Methods section is available in the online-only Data Supplement.

### Co-Immunoprecipitation Using Magnetic Dynabeads

3.25×10^7^ Dynabeads (Life Technologies) were separately coated with 6 μg/mL of the following antibodies: (1) anti-eNOS (NOS3 A9; Santa Cruz Biotech); (2) eNOS isotype control (IgG2a Clone DAK-GO5; Dako); (3) anti-PlAP antibody (NDOG2); and (4) PlAP isotype control (IgG1 Clone MOPC-21; BioLegend). STBMV or STBEX pooled from 4 NP (1 mg/mL) were incubated with anti-human Fc receptor blocking reagent (10 μL; Miltenyi Biotec) for 10 minutes at 4°C to block any nonspecific antigen binding (same pool was used at each experiment). Next, antibody-coated Dynabeads were incubated overnight at 4°C with 25 μg of protein from either STBMV (≈2.14×10^9^ EV/mL) or STBEX (≈3.2×10^9^ EV/mL) pools in 1 mL filtered PBS, as per manufacturer’s instructions. STBMV or STBEX bound to antibody-coated Dynabeads were separated and washed with PBS using a magnetic particle concentrator (Dynal MPC-S; ThermoFisher) and processed for Western blotting. See Methods section is available in the online-only Data Supplement for percentage calculations.

### Dimerization Analysis

To investigate eNOS dimerization in STBMV and STBEX, low-temperature SDS-PAGE was performed as previously described.^25^ NP samples at 15 μg were loaded under nonreducing conditions onto a 4% to 12% SDS-PAGE gel and run at 5 mA overnight at 4°C. Separated proteins were then transferred onto polyvinylidene difluoride membranes and blocked with 3% wt/vol BSA dissolved in Tris-buffered saline and Tween 20 for 1 hour prior to overnight incubation at 4°C with 0.6 μg/mL of anti-eNOS (NOS3-A; Santa Cruz) diluted in blocking buffer. Protein visualization was then performed on Western blot as described earlier.

### NOS Activity Assay

NOS activity by STBMV and STBEX was determined using an ultrasensitive colorimetric NOS assay kit, which converts all NO metabolites to nitrite using nitrate reductase (NB78 and NB70; Oxford Biomedical Research). We followed the manufacturer’s protocol and depicted nitrite accumulation as NOS activity (ie, production of NO). STBMV and STBEX pools of 4 NP patients (different pools used at each experiment) were first incubated in different concentrations (0, 10, 25, 50, and 100 μg). NOS activity was also measured with or without specific eNOS inhibitor, *N*^G^-nitro-l-arginine methyl ester (L-NAME; 1 mmol/L; Sigma-Aldrich), and the highly specific iNOS inhibitor, *N*-(3-(aminomethyl) bezyl) acetamidine) (2 μmol/L; Enzo Life Sciences).^26^ Inhibitors were incubated with STBMV and STBEX for 1 hour prior to analysis. Reactions were measured in triplicate, and absorbance was read at 540 nm.

### Statistical Analysis

The data were analyzed using GraphPad 5 software. Normality testing was performed using the Kolmogorov–Smirnov test and visual observation. The data were analyzed using paired or unpaired *t* test with Welch’s correction or 1-way analysis of variance, and data were presented as mean±SEM.

## Results

### Isolated STBEV Confirmed Microvesicular and Exosomal Phenotype

We first reconfirmed the expected microvesicular and exosomal phenotypes and sizes for STBEV (see Figure S1 in the online-only Data Supplement). Immunoblots of STBEV showed an enrichment of the STBEV marker, PlAP, relative to placental lysates. This enrichment was seen in both STBMV and STBEX (Figure S1A). STBEX were enriched for the exosomal markers ALIX, Syntenin, and CD9 (Figure S1A). Freshly isolated STBEV were analyzed on nanoparticle tracking analysis for size and particle number profiles. STBMV showed modal size of 323.2±7.1 nm with a broad size distribution, while STBEX were smaller (189.3±9.7 nm) with narrower size distribution (Figure S1B). Representative transmission electron micrographs confirmed the nanoparticle tracking analysis, and Western blot data showing STBMV was enriched for heterogeneous EV>200 nm, while STBEX contained a more homogenous population with a range of 30 to 200 nm EV (Figure S1C). These data confirmed that STBMV and STBEX had been successfully isolated.

### Syncytiotrophoblast and STBEV Express eNOS but Not iNOS

**Figure 1.**
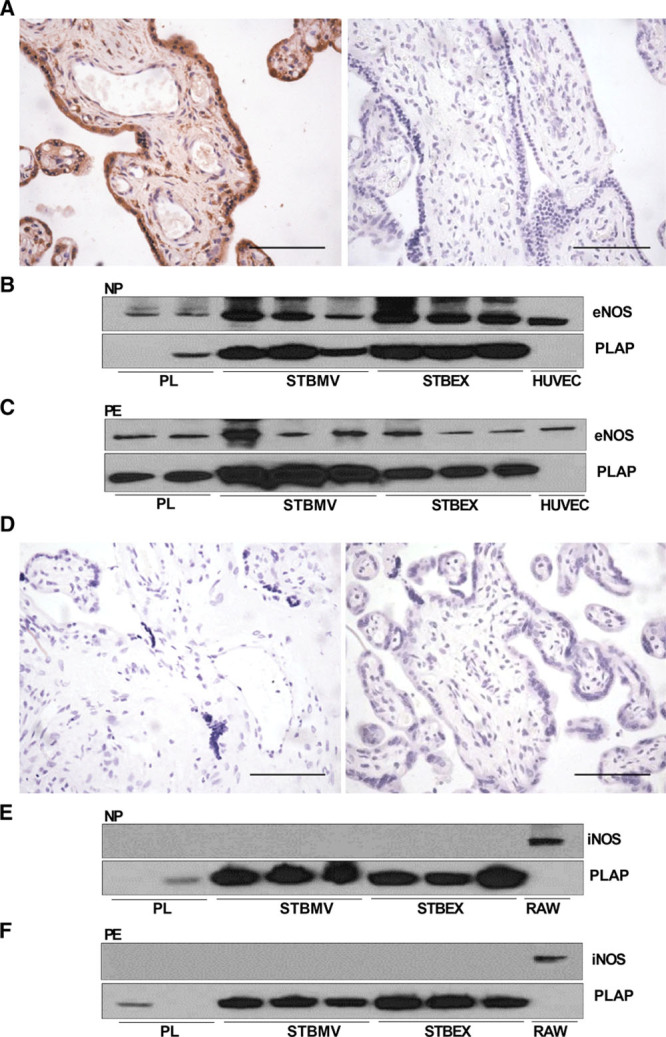
Immunohistochemical staining of normal pregnancy (NP) placenta tissue and Western blot of NP and preeclampsia (PE)-derived placental lysate (PL), syncytiotrophoblast extracellular microvesicles (STBMV) and syncytiotrophoblast extracellular exosomes (STBEX; n=3). **A**, Placental tissue showing endothelial nitric oxide synthase (eNOS) staining (brown; **left**) on syncytiotrophoblast (STB) layer and IgG2a-negative staining (**right**). Immunoblot showing eNOS (140 kDa) and placental alkaline phosphatase (PlAP; 60 kDa) expression in PL, STBMV, and STBEX derived from NP (**B**) and PE (**C**) similar to expression of positive control, human umbilical vein endothelial cells (HUVEC). **D**, Placental tissue demonstrating lack of inducible nitric oxide synthases (iNOS) staining (**left**) and isotype control IgG1-negative staining (**right**). Immunoblot showing no expression for iNOS (131 kDa) though positive control, RAW, is expressed and PlAP (60 kDa) is also expressed in PL, STBMV, and STBEX derived from NP (**E**) and PE (**F**). Scale bar set at 100 mm.

Immunohistochemical analysis of placental tissue revealed eNOS expression primarily localized in the syncytiotrophoblast cell layer of NP samples in comparison to negative staining of isotype control IgG2a (n=3; Figure 1A), confirming findings made previously by others.^13–15^ Similarly, eNOS expression was also shown in the placental lysates. We were also able to visualize eNOS expression in STBMV and STBEX isolated from NP (Figure 1B) and from preeclampsia (Figure 1C) placentae. iNOS expression was not detectable in the syncytiotrophoblast cell layer (n=3; Figure 1D). Additionally, no expression of iNOS in either placental lysates, STBMV, or STBEX from NP (Figure 1E) or preeclampsia placentas (Figure 1F) was detected (n=3). Nevertheless, expression of iNOS was seen in positive control RAW 264.7 cells, reassuring the antibody’s quality (Figure 1E and 1F). Positive PlAP expression indicated that the EV analyzed were derived from syncytiotrophoblast. The absence of iNOS prompted us to perform mass spectrometry.

### Mass Spectrometry Data

We assessed a mass spectrometry analysis we have performed on placental lysate, STBMV and STBEX from NP (n=6) and preeclampsia (n=8) patients (Tannetta et Al, unpublished data), and specifically interrogated it for the presence of NOS isoforms (see Table S1). The mass spectrometry was clearly able to identify eNOS, but iNOS was not identified at the protein level. Given these confirmatory findings, we, therefore, focused on STBEV expressing eNOS.

### Flow Cytometric Analysis of Ex Vivo and In Vivo–Derived STBMV Reveal Coexpression of eNOS and PlAP

**Figure 2.**
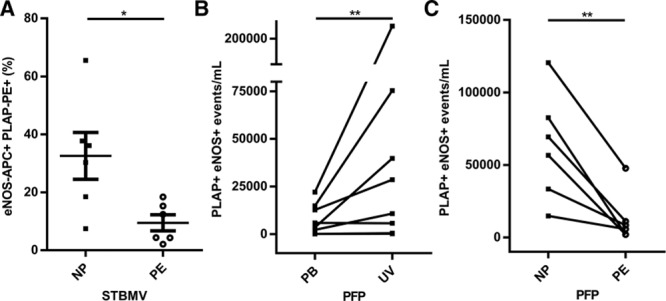
Flow cytometry analysis of ex vivo syncytiotrophoblast extracellular microvesicles (STBMV) derived from normal pregnancy (NP) and preeclampsia (PE) placentae (n=6). **A**, STBMV-bound endothelial nitric oxide synthase (eNOS) expression were significantly lower in PE compared with NP (*P<0.05). Flow cytometry analysis of circulating in vivo STBMV derived from paired peripheral vein blood (PB) and uterine vein blood (UV) plasma (n=8). **B**, Double-positive eNOS and placental alkaline phosphatase (PlAP) events per milliliter were higher in UV compared with PB (**P<0.01). Flow cytometry analysis of circulating in vivo syncytiotrophoblast extracellular vesicles (STBEV) derived from matched PB plasma of NP and PE patients (n=6). **C**, PlAP and eNOS double-positive STBMV events per milliliter were significantly lower in plasma from PE compared with NP (**P<0.01). PFP indicates platelet-free plasma.

STBMV derived from NP and preeclampsia placental perfusions were analyzed by flow cytometry for eNOS and PlAP coexpression (n=6). Using the corresponding fluorescence minus one controls, our data revealed double eNOS and PlAP positivity of 30.3±8.5% in NP-derived STBMV and 6.3±3.3% in preeclampsia-derived STBMV (*P<0.05; Figure 2A).

Circulating STBMV were also analyzed by flow cytometry in plasma prepared from paired PB and UV samples (n=8). PlAP and eNOS double-positive events per milliliter were significantly higher in UV (49 686±28 162) compared with PB (7723±2823) plasma samples, confirming STBMV-bound eNOS consistent with their origin from the placenta (**P<0.01; Figure 2B).

Similarly, STBMV-bound eNOS was measured in gestational age–matched plasma PB samples from NP and preeclampsia patients (n=6). After exclusion of EV derived from other cells rather than the syncytiotrophoblast, the results showed a significant reduction in PlAP and eNOS double-positive events per milliliter of plasma-derived STBMV from preeclampsia (12 798±7121) compared with NP (62 838±15 246; **P<0.01; Figure 2C). These data suggest that there is less plasma-derived STBMV-bound eNOS in preeclampsia compared with NP.

### eNOS and PlAP Are Expressed on the Same EV Population

Immunodepletion was used to further confirm STBMV and STBEX coexpression of eNOS and PlAP and exclude potential aggregation.

**Figure 3.**
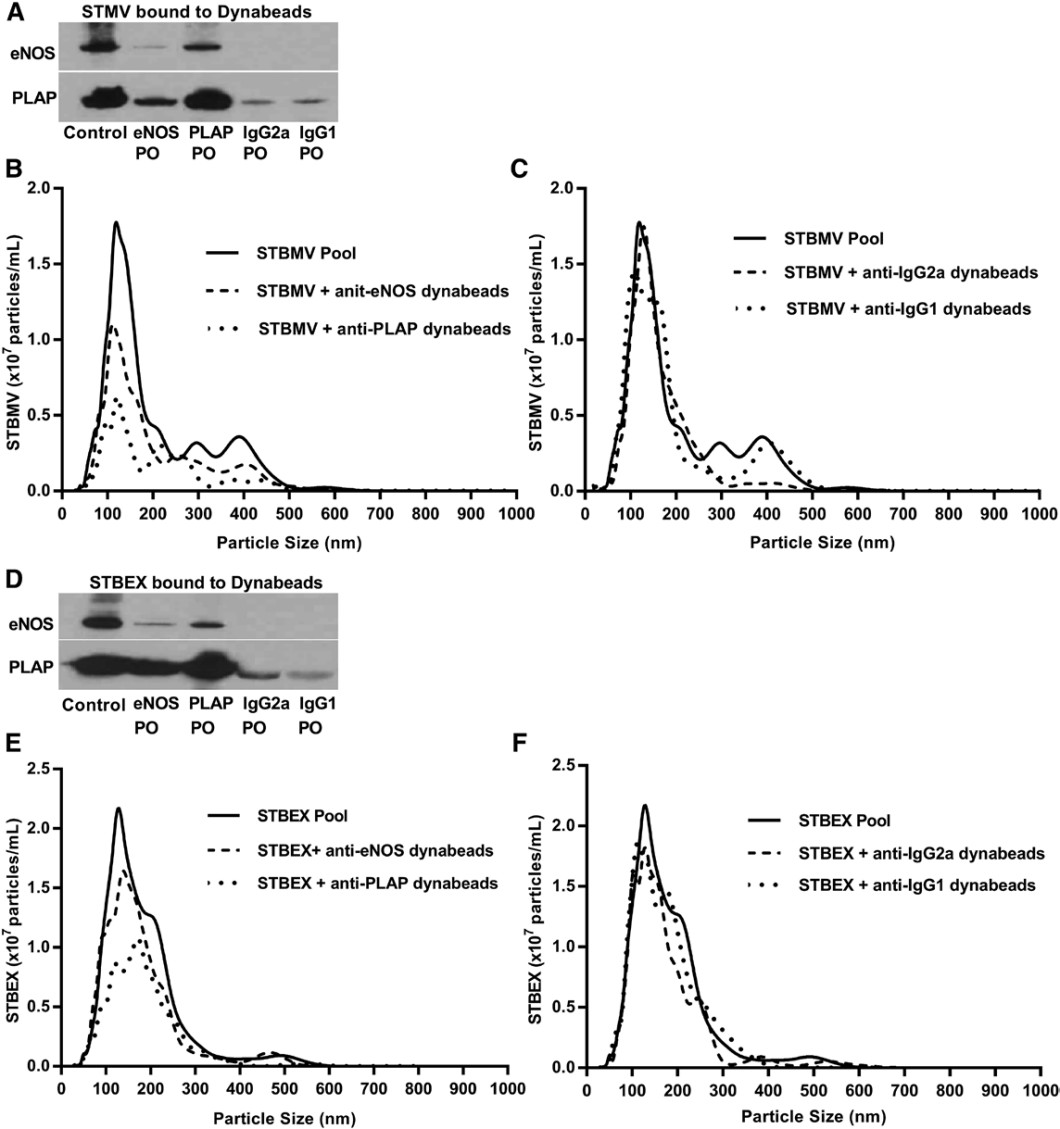
Immunobead depletion and nanoparticle tracking analysis (NTA) profiles of syncytiotrophoblast extracellular microvesicles (STBMV) and syncytiotrophoblast extracellular exosomes (STBEX) pools from normal pregnancy (NP; n=3). **A**, Representative immunoblot showing endothelial nitric oxide synthase (eNOS) and placental alkaline phosphatase (PlAP) coexpression on STBMV pool (Total) and STBMV pulled out (PO) with anti-eNOS Dynabeads, anti-PlAP Dynabeads, anti-IgG2a Dynabeads (isotype control for eNOS), and anti-IgG1 Dynabeads (isotype control for PlAP). **B**, Representative NTA size vs number profiles of STBMV pool (solid line), supernatant from post-incubation with anti-eNOS Dynabeads (dashed line) and anti-PlAP Dynabeads (dotted line). **C**, STBMV pool (solid line), supernatant of post incubation with anti-IgG2a Dynabeads (dashed line) and anti-IgG1 Dynabeads (dotted line). **D**, Representative immunoblot showing eNOS and PlAP coexpression on STBEX pool (Total) and STBEX PO with anti-eNOS, anti-PlAP, anti-IgG2a, and anti-IgG1 Dynabeads. **E**, Representative NTA size vs number profiles of STBEX pool alone (solid line), supernatant from post-incubation with anti-eNOS (dashed line) and anti-PlAP (dotted line) Dynabeads. **F**, STBEX pool (solid line), supernatant of post incubation with anti-IgG2a (dashed line) and anti-IgG1 (dotted line) Dynabeads.

For Western blot analysis, we used anti-eNOS–coated beads to immunoprecipitate eNOS-positive STBEV, which were interrogated for the presence of PlAP. We also conversely used anti-PlAP–coated beads to immunoprecipitate PlAP-positive STBEV, which were then interrogated for eNOS. Figure 3A reveals that anti-eNOS–coated magnetic beads could pull out STBMV-bound eNOS, which were positive for PlAP. The converse experiment using anti-PlAP–coated beads revealed similar results, confirming that both species were bound on the same population of STBMV (Figure 3A). Interestingly, PlAP pull out yielded a greater signal for eNOS compared with the eNOS pull out, which may indicate that some eNOS is intravesicular. To assess the relative quantities of STBMV-bound eNOS or PlAP, we used nanoparticle tracking analysis. The total concentration of STBMV was compared with the supernatant samples from the bead depletion experiments. This data showed that 35.5±4.2% of STBMV were eNOS positive and 64.3±0.4% were PlAP positive (n=3; Figure 3B), while IgG2a control was positive for 11.3±5.7%, and IgG1 control was positive for 11.4±3.4% (n=3; Figure 3C).

Analysis of the STBEX fraction (Figure 3D) revealed similar results. STBEX-bound eNOS comprised 36.7±6.6% while STBEX-bound PlAP was 39.2±5.8% of the initial preparation (n=3; Figure 3E), while IgG2a control was positive for 10.8±4.5% and IgG1 control was positive for 15.1±5.8% (n=3; Figure 3F).

### STBEV-eNOS Is Functional and Produce NO

**Figure 4.**
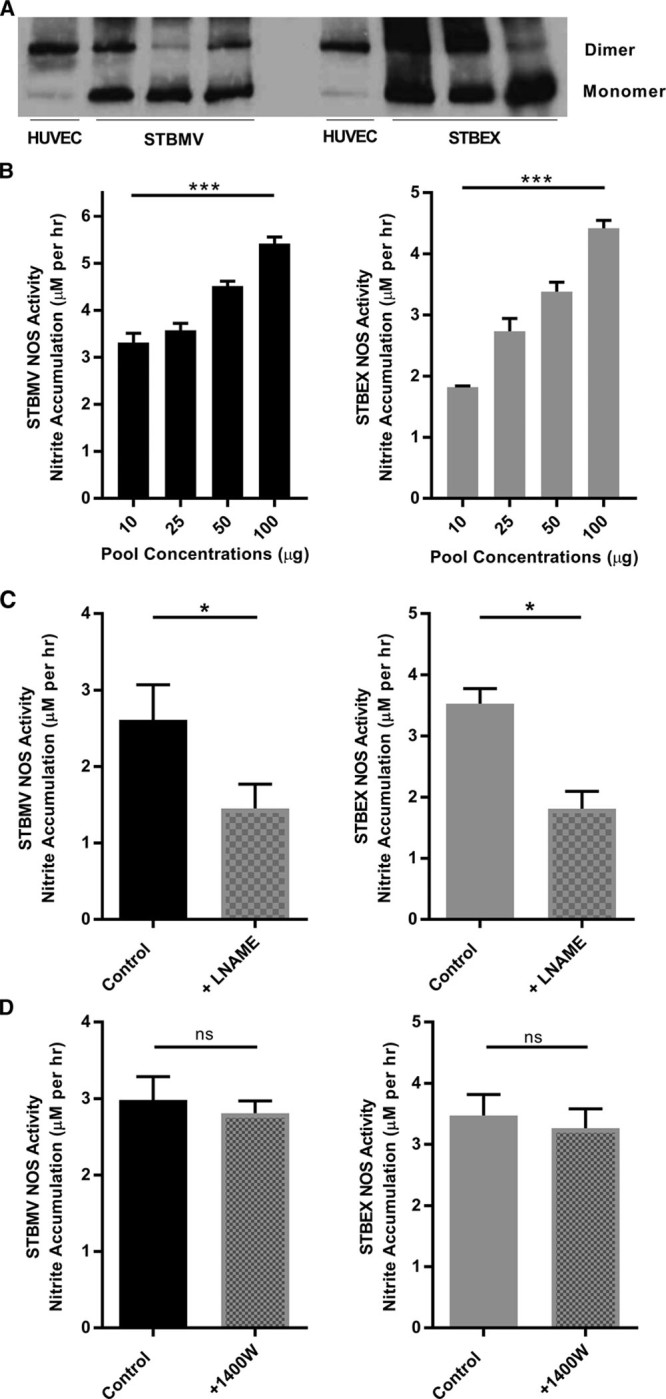
Syncytiotrophoblast extracellular microvesicles (STBMV) and syncytiotrophoblast extracellular exosomes (STBEX) express functional endothelial nitric oxide synthase (eNOS) and produce nitric oxide (NO). **A**, Dimerization of syncytiotrophoblast extracellular vesicles (STBEV) immunoblot image showing eNOS dimer (260 kDa) and eNOS monomer (140 kDa) expressed in human umbilical vein endothelial cells (HUVEC), STBMV, and STBEX (n=3). **B**, STBMV (***P<0.001) and STBEX (***P<0.001) pools showed NO production in a dose-dependent manner (n=6). **C**, 25 μg pool of STBMV and STBEX preincubated for 1 hour with 1 mmol/L of *N*^G^-nitro-l-arginine methyl ester (L-NAME), NOS inhibitor, showed significant reductions in NO production compared with controls (both *P<0.05; n=3). **D**, 25 μg pool of STBMV and STBEX preincubated for 1 hour with 2 μmol/L *N*-(3-(aminomethyl) bezyl) acetamidine) (1400W), inducible nitric oxide synthases (iNOS)–specific inhibitor, showed no changes in NO production compared with controls (both ns; n=3).

eNOS dimer (260 kDa) and monomer (140 kDa) were separated and visualized on Western Blot from ex vivo–derived STBMV and STBEX (n=3; Figure 4A), suggesting STBMV- and STBEX-bound eNOS are active. This was confirmed by demonstrating increasing dose-dependent NOS activity in terms of nitrite accumulation per hour (n=6 pooled STBEV; ***P<0.001; Figure 4B) as the protein concentration of STBMV and STBEX was increased. Nitrite accumulation was reduced in STBMV and STBEX incubated with L-NAME (an eNOS inhibitor) compared with control (STBMV, *P<0.05; STBEX, *P<0.05, n=3; Figure 4C). STBMV and STBEX incubated with selective iNOS inhibitor *N*-(3-(aminomethyl) bezyl) acetamidine) (2 μM) showed no change compared with control (both ns, n=3; Figure 4D).

### Ex Vivo STBEV-eNOS Reduced Activity in Preeclampsia

**Figure 5.**
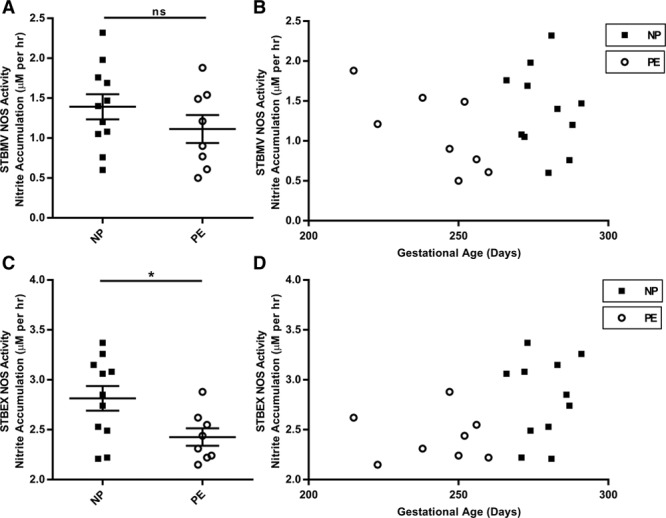
Nitric oxide synthase (NOS) activity of syncytiotrophoblast extracellular microvesicles (STBMV) and syncytiotrophoblast extracellular exosomes (STBEX) isolated from perfused normal pregnancy (NP) and preeclampsia (PE) placentae (n=11 and n=8, respectively). STBMV (**A**) from PE placentae showed a no overall reduction in NO production compared with NP (ns; *P*=0.2416). Analysis of the same data as a function of the gestational age of the samples (**B**) shows a decrease in NO production in samples closest in gestational age to controls (PE>34 weeks vs NP<40 weeks; *P<0.05). While STBEX (**C**) from PE showed a significant reduction in NO production compared with NP (*P<0.05). This decrease was maintained in samples closest in gestational age to controls (**D**).

NOS activity was compared between STBEV from NP (n=11) and preeclampsia (n=8) samples. For STBMV, the overall data showed no statistical difference in NOS activity (Figure 5A). When we analyze the same data taking into account gestational age (Figure 5B), we are able to see that preeclampsia samples from patients with a gestational age most similar to the available controls show a reduction in NOS activity (>34 weeks preeclampsia versus <40 weeks NP samples; *P<0.05). The earlier gestational ages are more difficult to interpret because of the lack of age-matched NP controls.

The STBEX showed an overall significant reduction in NOS activity (*P<0.05; Figure 5C), which persisted when we compared preeclampsia samples from patients with a gestational age most similar to controls (Figure 5D).

Taking STBMV and STBEX together, there is an overall reduction in NOS activity in preeclampsia compared with control.

## Discussion

We have demonstrated for the first time that eNOS is coexpressed with PlAP, the syncytiotrophoblast marker, on both STBMV and STBEX isolated from ex vivo NP placentas by Western blot, flow cytometry, and paramagnetic immunoprecipitation. Ex vivo–derived STBMV- and STBEX-bound eNOS exists as dimers, a feature required for NOS catalytic activity,^27^ and confirmed that these are capable of producing NO, which can be inhibited by L-NAME (an eNOS inhibitor). In addition, flow cytometry evaluation of in vivo–derived circulating STBMV from matched PB and UV plasma revealed that STBMV-eNOS are released by syncytiotrophoblast and circulate in the maternal blood.

NO plays an important role in mediating NP vasodilation, while defective endothelial NO synthesis and bioavailability has been associated with preeclampsia.^28,29^ This is the first study to reveal ex vivo–derived STBMV and STBEX isolated from placental perfused lobes to have less eNOS activity in preeclampsia in comparison to controls. Similarly, in vivo–derived plasma STBMV analyzed by flow cytometry showed less STBMV-bound eNOS expression in preeclampsia compared with NP.

We were unable to observe iNOS expression in NP syncytiotrophoblast and its derived STBEV either by Western blotting or mass spectrometry. iNOS expression and activity has been described by some in syncytiotrophoblast and placenta lysate.^14,30,31^ iNOS differences between normal and hypertensive pregnancies have been reported,^32,33^ while other studies have reported the absence of iNOS in the syncytiotrophoblast^34,35^ and a debateable relationship between iNOS and preeclampsia.^36^ Nevertheless, we think eNOS is the principal isoform bound to STBMV and STBEX.

Several studies corroborate our findings. It has been reported that isolated EV from plasma exert various effects on endothelial and trophoblast cells according to the physiological/pathological state of the pregnant woman.^37,38^ A decrease in expression and activity of eNOS bound to circulating nonpregnant EV has also been associated with cardiovascular diseases.^39^ Kao et al^40^ showed a significant increase in superoxide levels and impaired endothelial dysfunction in rat uterine arteries incubated with human preeclampsia plasma. Interestingly, L-NAME abolished this uterine artery vasodilation in both NP- and preeclampsia-derived plasma-treated vessels, suggesting that this effect was because of eNOS. Their findings support ours, although circulating factors rather than STBEV were described.

We hypothesize that decreased systemic eNOS in the form of circulating STBEV-eNOS may contribute to the reduced bioavailability of NO seen in preeclampsia, possibly affecting vascular functions. We are uncertain as to whether eNOS is donated to endothelial cells or whether NO is produced close to the endothelial cell and work to elucidate this is ongoing. We therefore interpret our data with some caveats. Although we show decreased NO production by STBEV-eNOS, there may also be a few other factors that contribute to the reduction of NO bioavailability. There may be (1) variation in NOS gene expression and activity; (2) lower substrate levels for NOS; (3) elevated inhibitors of NOS; or (4) increased breakdown of NO per se.^41^ Decreased NO release would not necessarily be limited by the shedding of less STBEV-eNOS from placenta. Other cell types expressing eNOS, such as platelets,^42^ endothelial progenitor cells,^43^ or circulating EV derived from endothelial cell and red blood cell,^39^ may contribute to overall NO bioavailability.

Our study has some limitations. Exosomes cannot be readily examined by routine flow cytometry because their size is below the limit of resolution. Thus, we were unable to measure STBEX-bound eNOS expression as we did with STBMV. Notwithstanding, we did see decreased levels of NO production in preeclampsia-derived STBEX. It is difficult to age match placental tissue in a disease which typically occurs earlier in pregnancy. We have attempted to mitigate this by using placentae from patients who developed preeclampsia later in pregnancy, but this limitation is common to all preeclampsia studies. Also, despite measuring STBMV-bound eNOS expression in plasma, attempts to measure NO production from plasma-derived STBMV and STBEX were unsuccessful because we were below the NOS assay limits of detection.

In conclusion, our data show that both STBMV and STBEX carry functionally active eNOS. Also, iNOS is not expressed in either STBMV or STBEX, thus, making STBEV-eNOS the principal moiety. Furthermore, STBEV-eNOS activity is reduced in preeclampsia, significantly in STBEX-bound eNOS. These findings suggest that STBEV-eNOS might contribute to the overall decreased NO bioavailability seen in preeclampsia.

## Perspectives

This study demonstrates that STBEV carry active eNOS into the maternal circulation. Importantly, the lower circulating STBEV-eNOS seen in preeclampsia might contribute to the diminished NO reported in this disease.

## Acknowledgments

We acknowledge Gavin Collet and Benedikt Kessler for generating the mass spectrometry data, Shijei Cai for his research insight, and research midwives Linda Holden and Fenella Roseman for recruiting patients for this study.

## Sources of Funding

This study was supported by an MRC Programme Grant (MR/J003360/1).

## Disclosures

None.

## Supplementary Material

## Novelty and Significance

### What Is New?

- Ex vivo and in vivo circulating–derived placental extracellular vesicles express functional endothelial nitric oxide synthase (eNOS), not inducible nitric oxide synthase.
- The plasma circulating levels of syncytiotrophoblast extracellular microvesicles–bound eNOS is significantly reduced in patients with preeclampsia compared with gestational age–matched controls.
- The circulating syncytiotrophoblast extracellular microvesicles–bound eNOS is produced by the placenta and elevated in uterine vein blood compared with peripheral blood.
- Ex vivo syncytiotrophoblast extracellular microvesicles and syncytiotrophoblast extracellular exosomes–bound eNOS activity is significantly decreased overall in preeclampsia compared with controls.

### What Is Relevant?

- Preeclampsia is a major cause of maternal morbidity and fetus mortality.
- Decreased circulating syncytiotrophoblast extracellular vesicles–bound eNOS might contribute to the overall decreased nitric oxide bioavailability seen in preeclampsia.
- Placental syncytiotrophoblast extracellular vesicles may play a role in signaling from the placenta to the maternal vascular system.

### Summary

The presence of trophoblast-derived circulating eNOS potentially links abnormal placental function to altered maternal vascular status.
