# Supplementary material for: Placental Vesicles Carry Active Endothelial Nitric Oxide Synthase and Their Activity is Reduced in Preeclampsia
Source: Hypertension. 2017 Jul 12;70(2):372–81. doi: 10.1161/HYPERTENSIONAHA.117.09321 (PMC5507817; doi:10.1161/HYPERTENSIONAHA.117.09321)
Supplement: Supplementary file 1 [file hyp-70-372-s001.docx]

**ONLINE SUPPLEMENTARY DATA**

**PLACENTAL VESICLES CARRY ACTIVE ENDOTHELIAL NITRIC OXIDE SYNTHASE AND THEIR ACTIVITY IS REDUCED IN PREECLAMPSIA**

**Authors**

Carolina Motta-Mejia ^1, 2^, Neva Kandzija ^1^, Wei Zhang ^1^, Vuyane Mhlomi ^1^, Ana Sofia Cerdeira ^1^, Alexandra Burdujan ^1^, Dionne Tannetta ^3^, Rebecca Dragovic ^1^, Ian L. Sargent ^1^, Christopher W. Redman ^1^, Uday Kishore ^2^ and Manu Vatish ^1^

^1^ Nuffield Department of Obstetrics & Gynaecology, University of Oxford, Women’s Centre, John Radcliffe Hospital, Oxford OX3 9DU, UK

^2^ Biosciences, College of Health and Life Sciences, Brunel University London, Uxbridge, UB8 3PH, UK

^3^ Department of Food and Nutritional Sciences, University of Reading, Reading
RG6 6AP, UK

**Short title:** Reduced eNOS activity in STBEV from PE patients

**Keywords:** preeclampsia, hypertension, nitric oxide, eNOS, syncytiotrophoblast extracellular vesicles

**Corresponding author:** Dr. Manu Vatish, MBBCh BA (Hons) DPhil MA MRCOG, Nuffield Department of Obstetrics & Gynaecology, University of Oxford, Women’s Centre, John Radcliffe Hospital, Oxford OX3 9DU, UK. Ph: +44 (0)1865 221009 Fax: +44 (0)1865 769141 Email: [manu.vatish@obs-gyn.ox.ac.uk](mailto:manu.vatish@obs-gyn.ox.ac.uk)

**SUPPLEMENTARY METHODS**

Cell Culture

HUVEC were isolated from NP umbilical cords, as previously described ^1^, grown at 37°C with 5% v/v CO_2_ using endothelial cell medium (EGM-2 SingleQuot Kit CC-4176, Lonza). Cell lysates were obtained with cold RIPA buffer, subjected to BCA protein assay and stored at -20°C.

Immunohistochemical Staining

Placental tissues were fixed in 4% v/v formaldehyde, embedded in paraffin blocks, cut in 8μm thick sections and placed on slides. Slides were deparaffinized in Histo-clear (Company), rehydrated in graded ethanol, and antigens were retrieved to enable detection using 0.01M Citrate buffer. Endogenous peroxidase was reduced with 3% v/v hydrogen peroxide. Tissue sections were blocked in 10% v/v fetal calf serum (FCS) for 1 h. Slides were incubated overnight at 4ºC with primary monoclonal antibodies against: eNOS (0.6 µg/mL, NOS3-A9 Santa Cruz), eNOS isotype control IgG2a (0.6 µg/mL, Clone DAK-GO5, Dako), iNOS (1 µg/mL, clone 2D2-B2, R&D) and iNOS isotype control IgG1 (1 µg/mL, IgG1 Clone MOPC-21, BioLegend in 1% v/v FCS in PBS with 0.01M Tween 20 (PBS-T) overnight at 4°C. All sections were then incubated with of 0.2 µg/mL anti-mouse IgG secondary antibody conjugated to horseradish peroxidase (HRP) in 10% v/v FCS for 1 h at room temperature. After washing with PBS-T, Antigen-specific detection was revealed using DAB kit (Vector laboratories) and counterstained with Shandon Gill 2 Haematoxylin (ThermoFisher). The slides were dehydrated in graded ethanol and mounted with Depex (VWR). Sections were viewed under a Leica DM2500 optical microscope (Leica Microsystems), and photographed using a digital camera linked to a computer hard drive (Micropublisher 5.0 RTV).

Flow Cytometry Analysis

Placental Perfusion Derived STBMV

Multi-colour flow cytometry panel was used to analyse placental perfusion derived STBMV. Flow cytometer and settings used for analysis were the same as previously described ^2, 3^, to allow comparison between different samples. Fluorochrome compensation was set using BD CompBeads (BD Biosciences), REA CompBeads (Miltenyi Biotech) and a single stain using Bio-Maleimide labelled ex vivo STBMV. STBMV were first incubated with 10 µL of FcR blocking reagent for 10 mins at 4ºC prior to labelling with antibodies and isotope matched control antibody for 15 mins at room temperature in the dark. The EV marker Bio-maleimide, which stains membrane proteins, was used to confirm biological material. CD41 and CD235a/b were used to exclude platelet and red blood cell (RBC) EV contamination, respectively. The STBEV specific marker PLAP) was used to confirm placental origin of the vesicles. Anti-human eNOS-APC conjugated was used to detect eNOS. The corresponding isotope controls were used as Fluorescence minus one (FMO) controls to discriminate true events from noise and set control gates. (Antibodies’ details in Online Data Supplement Table S1). Flow cytometry gating strategy was used to examine the data (see Online Data Supplement Figure S2).

Platelet Free Plasma (PFP) derived STBMV

Circulating STBMV from PB and UV were analysed by flow cytometry using a previously described protocol and flow cytometer settings ^4^, with additional modifications to exclude potential non-placental EV contaminants. Peripheral vein blood (PB) and uterine vein blood (UV) PFP samples were thawed in a water bath at 37 ºC. 200µl from each plasma sample was labelled with the ‘Dump Channel’ antibodies. ‘Dump Channel’ includes contaminant markers against Classical HLA class I and II (leukocytes), CD235a/b (RBC) and CD41 (platelet) all conjugated to PEvio770 (Miltenyi Biotec). Sample was also stained against PLAP-PE (STB marker) and eNOS-APC for co-expression analysis. (See Online Data Supplement Table S1 for antibodies’ details.) Plasma sample were incubated for staining for 15 min at 4ºC in the dark. Samples were passed through a durapore-PVDF 0.22µm filter (Ultrafree-MV-GV, Millipore) by centrifugation at 800g for 3 mins (5430R, Eppendorf) in order to concentrate the EV > 200 nm on the filter membrane. The filtrate (pass through) was topped up to 800 µL with sterile PBS (1/5 dilution). The EV (on the filter membrane) were carefully resuspended using 100 µL filtered PBS and stained in the dark with 10 µL Bio-maleimide for 10 min at room temperature. After staining, the EV sample was topped up with filtered PBS up to 500 µL (1/5 dilution), and then analysed for 10 min of data acquisition on the flow cytometer. EV sample was then incubated with 20 µL (1/25 dilution) of neat detergent Nonidet P-40 detergent (Sigma) for 20 min in the dark (to disrupt the vesicle membranes). The EV sample-treated with detergent, together with the filtrate (pass through), were also analysed for 2 min data acquisition on the flow cytometer and used to set up the control gates.

Plasma derived STBMV data was then analysed using the following gating strategy (see Online Data Supplement Figure S3). The filtrate sample (pass through) was used to set up the ‘Dump Channel’ control gate at 1% cut off to separate EV population positive for Bio-Maleimide and negative for ‘Dump Channel’ contaminants. The EV sample-treated with detergent was used to set up the PLAP+ eNOS+ control gates at 1% cut off. Once control gates were set up, plasma derived STBMV double positive (PLAP+ eNOS+) events/mL were calculated taking into account the dilution factor of EV sample (1/5) and to standardise all samples to 1mL. The following formula we used:

PLAP+ eNOS+ STBMV events/mL= (PLAP+ eNOS+ EV events X 5) X 5

Western Blotting

HUVEC lysate was used as a positive control for eNOS. Murine macrophage cells (RAW 264.7 cell lysate; Santa Cruz Biotech) were used as positive control for iNOS (30ug protein in all samples). (Antibody details below in Table S1). Reactions were visualized by using an appropriate secondary antibody conjugated to horseradish peroxidase (HRP) before incubation with HRP substrate enhanced luminescence (ThermoFisher).

Co- Immunoprecipitation using Magnetic Dynabeads

To calculate STBMV or STBEX particle number bound to antibody coated beads, STBMV or STBEX without Dynabeads (Total) and nominal unbound STBMV or STBEX fraction left in supernatant (eNOS and PlAP negative) were analysed using the NTA. The percentage of STBMV or STBEX bound eNOS or PlAP was calculated as follows: eNOS and PlAP positive = (([Total]-(eNOS and PlAP negative)) / [Total]) x 100.

**REFERENCES FOR SUPPLEMENETARY METHODS**

1. Jaffe EA, Nachman RL, Becker CG, Minick CR. Culture of human endothelial cells derived from umbilical veins. Identification by morphologic and immunologic criteria. J Clin Invest. 1973;52:2745-2756.

2. Dragovic RA, Southcombe JH, Tannetta DS, Redman CWG, Sargent IL. Multicolor flow cytometry and nanoparticle tracking analysis of extracellular vesicles in the plasma of normal pregnant and pre-eclamptic women. Biol Reprod. 2013;89.

3. Dragovic RA, Collett GP, Hole P, Ferguson DJ, Redman CW, Sargent IL, Tannetta DS. Isolation of syncytiotrophoblast microvesicles and exosomes and their characterisation by multicolour flow cytometry and fluorescence nanoparticle tracking analysis. Methods. 2015;87:64-74.

4. Inglis HC, Danesh A, Shah A, Lacroix J, Spinella PC, Norris PJ. Techniques to improve detection and analysis of extracellular vesicles using flow cytometry. Cytometry A. 2015;87:1052-1063.

**SUPPLEMENTARY TABLES**

| **Antibody/Dye** | **Concentration**  **/Dilution** | **Antigen** | **EV Specificity** | **Source** |
| --- | --- | --- | --- | --- |
| **Western Blot** |  |  |  |  |
| Anti-eNOS (NOS3-A9) | 0.6 µg/mL | eNOS | - | Santa Cruz |
| Anti-iNOS (clone 2D2-B2) | 1 µg/mL | iNOS | - | R&D |
| Anti-PLAP (NDOG2) | 0.6 µg/mL | PLAP | STBEV | In-house antibody |
| Anti-Alix | 1/1000 | Alix | Exosomes | Cell Signalling |
| Anti-Syntenin | 1/1000 | Syntenin | Exosomes | Abcam |
| Anti-CD9 | 1/200 | CD9 | Exosomes | Santa Cruz |
| Polyclonal goat-anti-mouse/rabbit immunoglobulin HRP | 1/2000 | Mouse/rabbit immunoglobulins | - | Cell Signalling |
|  |  |  |  |  |
| **Flow Cytometry** |  |  |  |  |
| STBMV Analysis |  |  |  |  |
| Bio-maleimide (BODIFY FL N-(-2-aminoethyl)-maleimide | 0.25µM | Thiol groups | All EV | Molecular probes |
| Anti-PLAP-PE (NDOG2) | 0.05µg/mL | PLAP | STBEV | In-house antibody |
| IgG1-PE (clone MOPC-21) | 0.05µg/mL | Isotype control | - | Biolegend |
| Anti-eNOS-APC (clone REA451) | 0. 4µg/mL | eNOS | - | Miltenyi Biotech |
| REA-APC | 0.025µg/mL | Isotype control | - | Miltenyi Biotech |
| Anti-CD41-PECy7 (clone P2) | 0.25µg/mL | CD41 | Platelet EV | Beckam Coulter |
| IgG1-PECy7 (clone MOPC-21) | 0.25µg/mL | Isotype Control | - | Beckam Coulter |
| Anti-CD235a/b-PECy5 (clone HIR2) | 0.05µg/mL | CD235a/b | RBC EV | Biolegend |
| IgG2b-PECy5 (clone MPC-11) | 0.05µg/mL | Isotype Control | - | Biolegend |
|  |  |  |  |  |
| Plasma Analysis |  |  |  |  |
| Bio-maleimide (BODIFY FL N-(-2-aminoethyl)-maleimide | 0.5µM | Thiol groups | All EV | Molecular probes |
| Anti-PLAP-PE (NDOG2) | 0.7µg/mL | PLAP | STBEV | In-house antibody |
| Anti-eNOS-APC (clone REA451) | 0.8µg/mL | eNOS | - | Miltenyi Biotech |
| Anti-CD41-PEvio770 | 0.33µg/mL | CD41 | Platelet EV | Miltenyi Biotech |
| Anti-CD235a/b-PEvio770 | 0.11µg/mL | CD235a/b | RBC EV | Miltenyi Biotech |
| Anti-HLA Class I-PEvio770 | 2.2mg/mL | HLA Class I | Leukocyte EV | Miltenyi Biotech |
| Anti-HLA Class II-PEvio770 | 0.165mg/mL | HLA Class II | Leukocyte EV | Miltenyi Biotech |
|  |  |  |  |  |

**Table S1.** Antibodies, fluorescent labels, isotype controls and secondary antibodies used for Western blot analysis, immunohistochemistry and flow cytometry.

| **NOS Isoforms** | **Samples** | **Peptide count** | **Unique peptides** | **Confidence Score** |
| --- | --- | --- | --- | --- |
| **eNOS** | PL | 3 | 3 | 159.32 |
|  | STBMV | 42 | 38 | 2476.18 |
|  | STBEX | 46 | 39 | 2757.70 |
| **nNOS** | PL | / | / | / |
|  | STBMV | 3 | 0 | 102.73 |
|  | STBEX | 2 | 0 | 73.98 |
| **iNOS** | PL | / | / | / |
|  | STBMV | / | / | / |
|  | STBEX | / | / | / |

**Table S2**. Nitric oxide synthases (NOS) isoforms identified by mass spectrometry analysis of placental lysate (PL), syncytiotrophoblast microvesicles (STBMV) and exosomes (STBEX) samples from 8 normal (NP) and 6 preeclamptic (PE) pregnancies. eNOS, endothelial nitric oxide synthase; nNOS, neuronal nitric oxide synthase; iNOS, inducible nitric oxide synthase.

**SUPPLEMENTARY FIGURES**

**C**

**A**


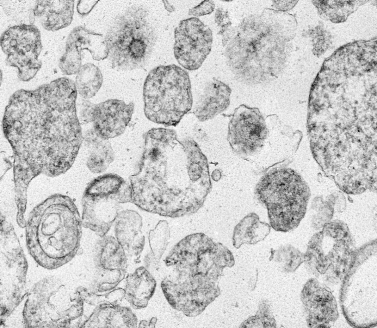

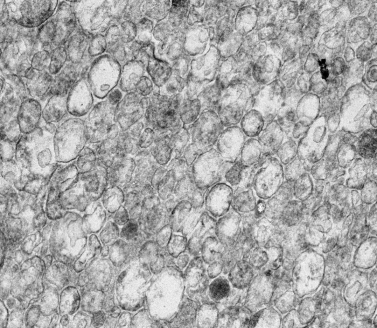

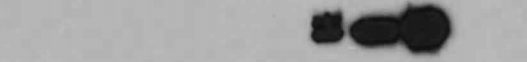


**PL**

**STBMV**

**STBEX**

**CD9**

**Synt**

**PLAP**

**Alix**


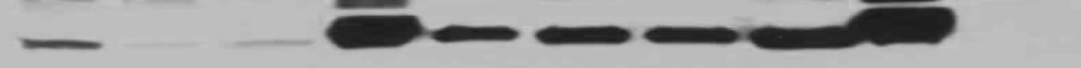


**B**

**STBMV**

**STBEX**

**Figure S1**. Characterisation of normal pregnancy (NP) derived syncytiotrophoblast microvesicles (STBMV) and exosomes (STBEX). **A**, Representative Immunoblot showing an enrichment of STBEV marker, PLAP (60 KDa) on NP placental lysates (PL) and STBEV; and an enrichment of exosomal markers Alix (96 KDa), Syntenin (60 KDa) and CD9 (24 KDa) on syncytiotrophoblast exosomes (STBEX). **B**, Representative Nanoparticle Tracking Analysis (NTA) size vs particle number distribution profiles of STBMV (323.2 ± 7 nm) and STBEX (189.3 ± 9.7 nm). **C**, Representative Transmission Electron Micrographs (TEM) of STBMV and STBEX. Scale bars represent 200 nm.

**A**

**B**

**D**

**E**

**F**

**G**

**H**

**I**

**SSC**

**CD235ab - PeCy5**

**PLAP – PE**

**eNOS - APC**

**CD41 - PeCy7**

**Bio-Maleimide - FITC**


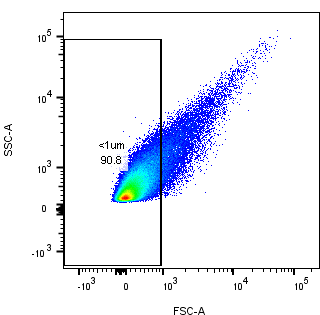

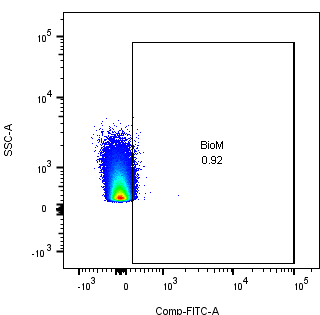


**SSC**

**FSC**


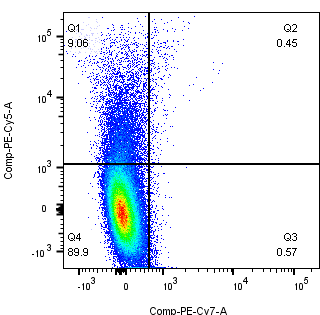

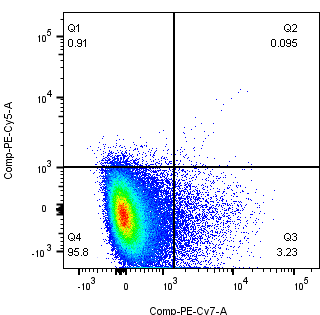

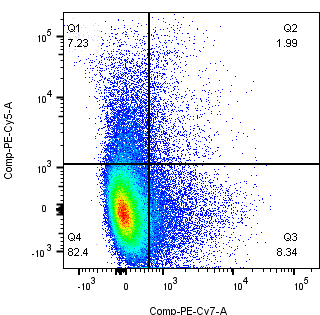

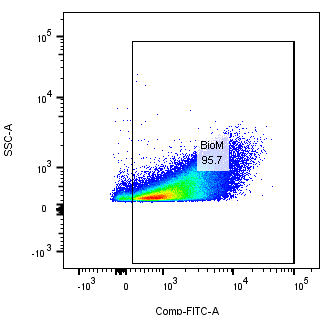

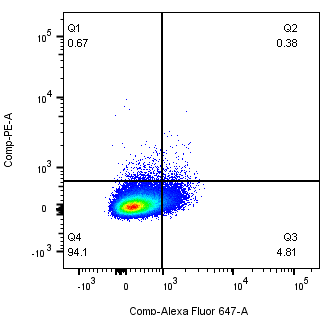


**C**


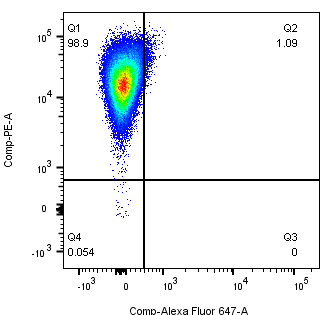

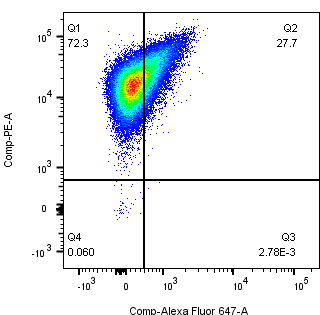


**Figure S2**. Representative flow cytometric multicolour gating strategy used to analyse STBMV. **A**, EV displayed on Forward Scatter (FSC) versus Side Scatter (SSC) plot with 1µm cut off gate., ≤ 1µm EV displayed on SSC vs Bio-maleimide (BioM) plot and stained with: (**B)** Bio- BioM fluorochrome minus one (FMO) to draw 1 % cut off gate for BioM positive; and (**C)**, BioM (EV marker) to include BioM positive EV population. CD235a/b-PECy5 vs CD41-PECy7 plots displaying ≤ 1µm BioM positive EV stained with: (**D**) CD235a/b FMO to draw 1% cut off gate for CD235a/b positive (Q1 and Q2); (**E**) CD41 FMO to draw the 1% cut off gate for CD41 positive (Q2 and Q3) and (**F)** CD235a/b and CD41 contaminant markers to exclude EV positive for CD235ab and CD41 (Q1, Q2 and Q3). ≤ 1µm BioM positive and negative for CD235a/b and CD41 EV displayed in a PLAP-PE vs eNOS-APC plot stained with: (**G**) PLAP FMO to draw 1% cut off gate for PLAP positive (Q1 and Q2); (**H**) eNOS FMO to draw 1% cut off gate for eNOS positive (Q2 and Q3) and (**I**) PLAP (STBMV maker) and eNOS (antigen of interest marker) to show final analysis of double positivity for PLAP and eNOS (Q2) on STBMV.


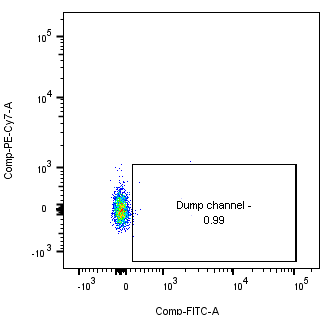

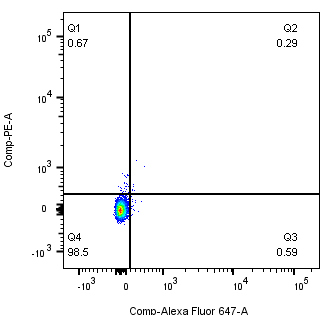

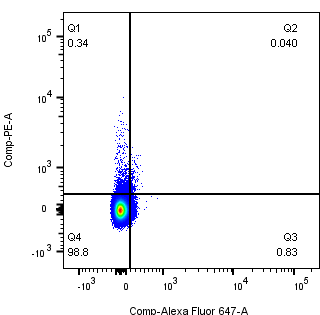


**A**

**B**

**C**

**D**

**Bio-Maleimide - FITC**

**eNOS - APC**

**PLAP - PE**

**Dump Channel- PeCy7**


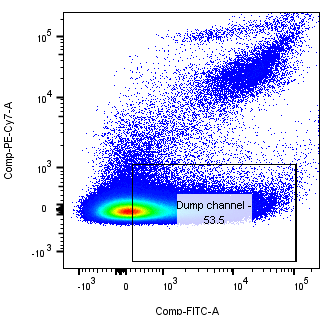


**EV sample**

**EV filtrate sample**

**EV sample treated with detergent**

**EV sample**

**PLAP - PE**

**eNOS - APC**

**Dump Channel- PeCy7**

**Bio-Maleimide - FITC**

**Figure S3**. Representative flow cytometric multicolour gating strategy used to analyse STBMV derived from platelet free plasma (PFP). **A**, The filtrate sample (pass through) used to draw the ‘Dump Channel’ gate at 1% cut off using Dump channel-PeCy7 vs. Bio-Maleimide-FITC dot plot. Dump Channel includes contaminant markers such as CD231a/b, CD41, HLA-ABC and HLA Class II; all conjugated with PEvio770 labelling. **B**, Dump channel negative and Biomaleimide positive STBMV stained sample was treated with detergent to draw the 1% cut off gates for PLAP+ (Q1 and Q2) and eNOS+ (Q2 and Q3), using PLAP-PE vs eNOS-APC dot plot. **C**, ‘Dump Channel’ positive population from the STBMV sample was excluded (outside dump channel gate), while ‘Dump Channel’ negative EV and Bio-Maleimide positive EV was included (inside dump channel gate). **D**, STBMV population double positive for PLAP and eNOS (Q2) showed circulating plasma derived STBMV co-expressed PLAP and eNOS. STBMV event number per mL was calculated.
